# Goldenseal (*Hydrastis canadensis* L.) Extracts Inhibit the Growth of Fungal Isolates Associated with American Ginseng (*Panax quinquefolius* L.)

**DOI:** 10.3390/molecules29030556

**Published:** 2024-01-23

**Authors:** Ying Gao, Ethan Swiggart, Kaela Wolkiewicz, Prabha Liyanapathiranage, Fulya Baysal-Gurel, Farhat A. Avin, Eleanor F. P. Lopez, Rebecca T. Jordan, Joshua Kellogg, Eric P. Burkhart

**Affiliations:** 1 International Ginseng Institute, School of Agriculture, Middle Tennessee State University, 1301 E Main St, Murfreesboro, TN 37132, USAklw2at@mtmail.mtsu.edu (K.W.); 2 Otis Floyd Nursery Research Center, Department of Agricultural and Environmental Sciences, Tennessee State University, 472 Cadillac Ln, McMinnville, TN 37110, USA; pliyanap@tnstate.edu (P.L.); fbaysalg@tnstate.edu (F.B.-G.);; 3 Soil, Plant and Pest Center, University of Tennessee, 5201 Marchant Dr, Nashville, TN 37211, USA; 4 Department of Veterinary and Biomedical Science, The Pennsylvania State University, University Park, PA 16802, USA; rtj5@psu.edu (R.T.J.); jjk6146@psu.edu (J.K.); 5 Shaver’s Creek Environmental Center, The Pennsylvania State University, 3400 Discovery Road, Petersburg, PA 16669, USA; 6 Department of Ecosystem Science and Management, The Pennsylvania State University, University Park, PA 16802, USA

**Keywords:** *Alternaria panax*, *Fusarium sporotrichioides*, *Pestalotiopsis nanjingensis*, antifungal, botanical pesticide

## Abstract

American ginseng, a highly valuable crop in North America, is susceptible to various diseases caused by fungal pathogens, including *Alternaria* spp., *Fusarium* spp., and *Pestalotiopsis* spp. The development of alternative control strategies that use botanicals to control fungal pathogens in American ginseng is desired as it provides multiple benefits. In this study, we isolated and identified three fungal isolates, *Alternaria panax*, *Fusarium sporotrichioides*, and *Pestalotiopsis nanjingensis*, from diseased American ginseng plants. Ethanolic and aqueous extracts from the roots and leaves of goldenseal were prepared, and the major alkaloid constituents were assessed via liquid chromatography–mass spectrometry (LC–MS). Next, the antifungal effects of goldenseal extracts were tested against these three fungal pathogens. Goldenseal root ethanolic extracts exhibited the most potent inhibition against fungal growth, while goldenseal root aqueous extracts and leaf ethanolic extracts showed only moderate inhibition. At 2% (*m*/*v*) concentration, goldenseal root ethanolic extracts showed an inhibition rate of 86.0%, 94.9%, and 39.1% against *A. panax*, *F. sporotrichioides*, and *P. nanjingensis*, respectively. The effect of goldenseal root ethanolic extracts on the mycelial morphology of fungal isolates was studied via scanning electron microscopy (SEM). The mycelia of the pathogens treated with the goldenseal root ethanolic extract displayed considerable morphological alterations. This study suggests that goldenseal extracts have the potential to be used as a botanical fungicide to control plant fungal diseases caused by *A. panax*, *F. sporotrichioides*, or *P. nanjingensis.*

## 1. Introduction

American ginseng (*Panax quinquefolius* L.) is a well-known perennial medicinal plant indigenous to forests in eastern North America. For a century, it has been cultivated commercially and produced as a specialty crop using several agricultural approaches, including using artificial shade in field cultivation (i.e., grown under polypropylene shade cloth) and forest farming (i.e., grown under the forest canopy) [1,2]. Fungal pathogens, including *Alternaria*, *Fusarium*, *Phytophthora*, *Cylindrocarpon, Pestalotiopsis*, and *Pythium*, are known to cause diseases and can cause severe losses in the production of this crop [3]. Infection by these pathogens causes many symptoms, such as damping off, root rot, rusty roots, or the abortion of developing berries, which negatively impact crop yield [2,3]. *Alternaria panax*, which is widely recognized as the main causal agent of the annual leaf blight problem faced by ginseng growers, results in premature defoliation if left uncontrolled [3,4,5]. Several studies reported the occurrence of Alternaria blight caused by *Alternaria panax* in American ginseng in Oregon, Washington, Wisconsin, Ontario, and British Columbia [4,5]. Bi et al. (2011) reported the occurrence of root rot caused by *Fusarium solani* or *F. oxysporum* in American ginseng in China [6]. Liyanapathiranage et al. (2023) reported the occurrence of leaf spots caused by *Pestalotiopsis nanjingensis* in American ginseng in the U.S. [7]. Punja et al. (2007) reported that a range of *Fusarium* species, including *F. equiseti*, *F. sporotrichioides*, *F. avenaceum*, *and F. culmorum*, caused the discoloration of ginseng roots in British Columbia [8]. These studies demonstrate that American ginseng is susceptible to diseases caused by the pathogens *Alternaria*, *Fusarium*, and *Pestalotiopsis*.

Conventionally, growers have relied on frequent applications of synthetic fungicides to protect their American ginseng crop. These are costly and may result in unintended consequences by inducing pathogen resistance [2,9]. In addition, the overuse of synthetic fungicides has created environmental problems. Recently, attention has been paid to the exploitation of botanical pesticides for crop protection [10]. The development of alternative control strategies using botanicals provides several benefits for controlling plant fungal pathogen diseases. Botanical pesticides not only reduce dependency on synthetic fungicides but may also be more eco-friendly [11]. For this reason, they are known as “green pesticides” [12]. Since botanical pesticides are made from extracts or essential oils from plants, they may be less toxic and degrade more quickly in the environment [11]. Botanical pesticides may contain a combination of antimicrobial compounds, making them effective against a range of plant pathogens with a lower risk of developing pathogen resistance as compared to synthetic pesticides [13]. Botanical pesticides may also be more affordable than synthetic fungicides, making them a cost-effective alternative. The demand for green pesticides has increased dramatically due to consumers’ preferences for organic food and nutraceutical products [12].

Goldenseal (*Hydrastis canadensis* L., Ranunculaceae/Hydrastidaceae) is a North American native medicinal plant that is often associated with the same forest habitat and production requirements as American ginseng in eastern North America [1,14,15]. Like ginseng, it is also a valuable specialty crop that is grown under artificial shade in field conditions or forest farmed on existing forestlands [1,16]. Goldenseal is commonly used as a natural remedy for a variety of purposes; the roots and rhizomes of goldenseal have documented antimicrobial properties and are used in herbal medicine to treat inflammation [17,18], while leaf extracts have been shown to possess antimicrobial activity against *Staphylococcus aureus* (MRSA) for skin infections [19]. There is evidence to suggest that goldenseal extracts have antifungal activity against certain pathogenic fungi that cause infections in humans [20]. Riešutė et al. (2022) reported the antifungal activity of goldenseal aqueous extracts on pathogenic yeast *Candida* cultures [21]. It is known that the antimicrobial property of goldenseal is attributed to three major alkaloids: berberine, hydrastine, and canadine [22,23,24]. However, crude plant extracts from goldenseal were shown to have greater antimicrobial activity than pure berberine, suggesting that other constituents enhance the efficacy of berberine [19].

In theory, goldenseal extracts could be used to prevent diseases in American ginseng caused by fungal pathogens. In addition to its demonstrated antimicrobial activity, there are three additional reasons for investigating goldenseal as an on-farm disease management resource for ginseng growers. First, goldenseal has inter- or relay-cropping potential with ginseng, especially in forest farming agroforestry systems. Some growers currently intercrop ginseng and goldenseal to increase crop diversity and revenue, while reducing pestilence, because the two species prefer similar cropping environments and both require multiple years to mature [1, Burkhart, personal observation]. Second, goldenseal has significantly lower wholesale value when compared with ginseng and may be available as a comparatively cost-effective disease management solution because it is already commonly grown with ginseng. For example, the wholesale market price paid for goldenseal is USD 20–40 per pound in recent decades compared with USD 100–1000 or more for ginseng [1]. And thirdly, goldenseal is easier to propagate than ginseng, as it can be propagated asexually and non-destructively via rhizome division. Once established, goldenseal can be harvested on an annual or semi-annual basis by removing rhizome sections, or aerial foliage [14], to prepare extracts as needed. This material may even be sourced following harvests using non-market raw materials (e.g., small root fragments and aerial foliage), making it a cost-effective and renewable on-farm resource for growers.

Despite this potential opportunity, there is little research on establishing appropriate protocols for optimal goldenseal extraction and determining the efficacy of this approach for disease control. The objectives of this study were twofold: first, to evaluate and standardize the methods of goldenseal extraction, and second, to assess the anti-fungal properties of goldenseal extracts against the three primary fungal isolates that affect American ginseng. To the best of our knowledge, this is the first study to explore the potential use of goldenseal extracts in fungal pathogen management for American ginseng. By providing growers with a botanical pesticide that could be developed on-farm for crop protection, our study could help increase the feasibility and profitability of organic ginseng production.

## 2. Results

### 2.1. Fungal Colony Morphology, Molecular Identification, and Pathogenicity Tests

The colony morphologies are described as follows: *Alternaria panax* exhibited slow growth, with a pale gray, cottony appearance and round margins, along with light brown mycelium. *Fusarium sporotrichioides* displayed fast growth, with orange-white, fluffy mycelia. *Pestalotiopsis nanjingensis* appeared as round, white, thick, and flocculent colony growth on the front of the plate and a yellowish ringed shape was observed on the back. The DNA sequencing and molecular identification of fungal isolates are listed in Supplementary Table S1.

For pathogenicity tests, one month after inoculation, plants inoculated with *P. nanjingensis* exhibited leaf spot symptoms that were previously identified in the initial symptomatic samples [6]. In contrast, plants inoculated with *A. panax* and *F. sporotrichioides* remained asymptomatic, similarly to control plants. 

### 2.2. The Extraction Rate of Ethanolic and Aqueous Extraction

Our study showed that sonication-assisted extraction was an efficient method of preparing extracts from goldenseal roots and leaves. As shown in Table 1, the two solvents (70% ethanol and diH_2_O) yielded a high extraction rate using sonication-assisted extraction, ranging from 22.6% to 34.6%.

### 2.3. Alkaloid Content in Aqueous and Ethanolic Extract of Goldenseal Root

**Figure 1:**
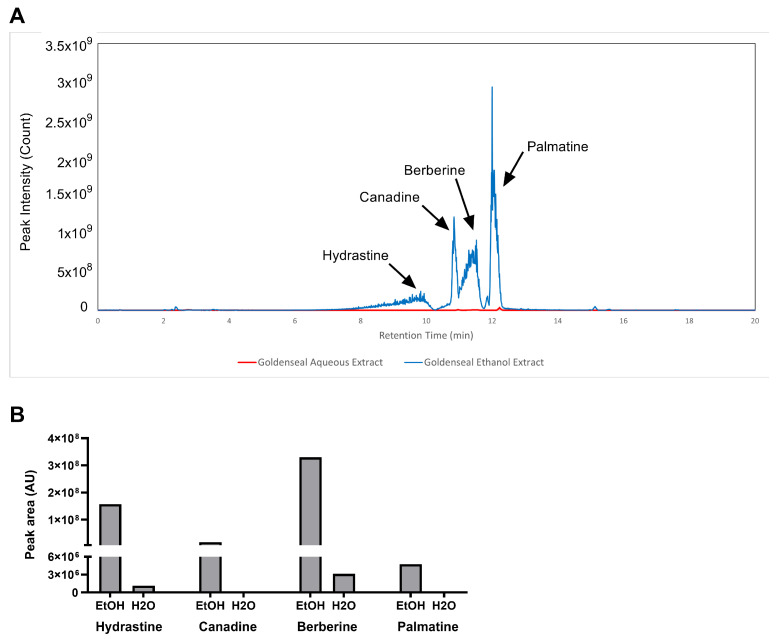
LC–MS analysis of alkaloid content in the ethanolic and aqueous extract of goldenseal root. (**A**) Chromatography of goldenseal root extracts. (**B**) Content of four alkaloids in goldenseal root extracts.

The aqueous and ethanolic extracts of goldenseal root qualitatively yielded similar chemical constituents, as observed in their chromatograms (Figure 1A). However, 70% ethanol was substantially more efficient at extracting protoberberine and isoquinoline alkaloids compared to water, most likely due to the enhanced solubility of these alkaloids in organic solvents. The ethanolic extract yielded peak areas for hydrastine, canadine, berberine, and palmatine that were 135, 517, 105, and 413 times greater than the corresponding peaks for the aqueous extract (Figure 1B).

### 2.4. Effect of Goldenseal Root Ethanolic Extract on the Growth of A. panax, F. sporotrichioides, and P. nanjingensis

**Figure 2:**
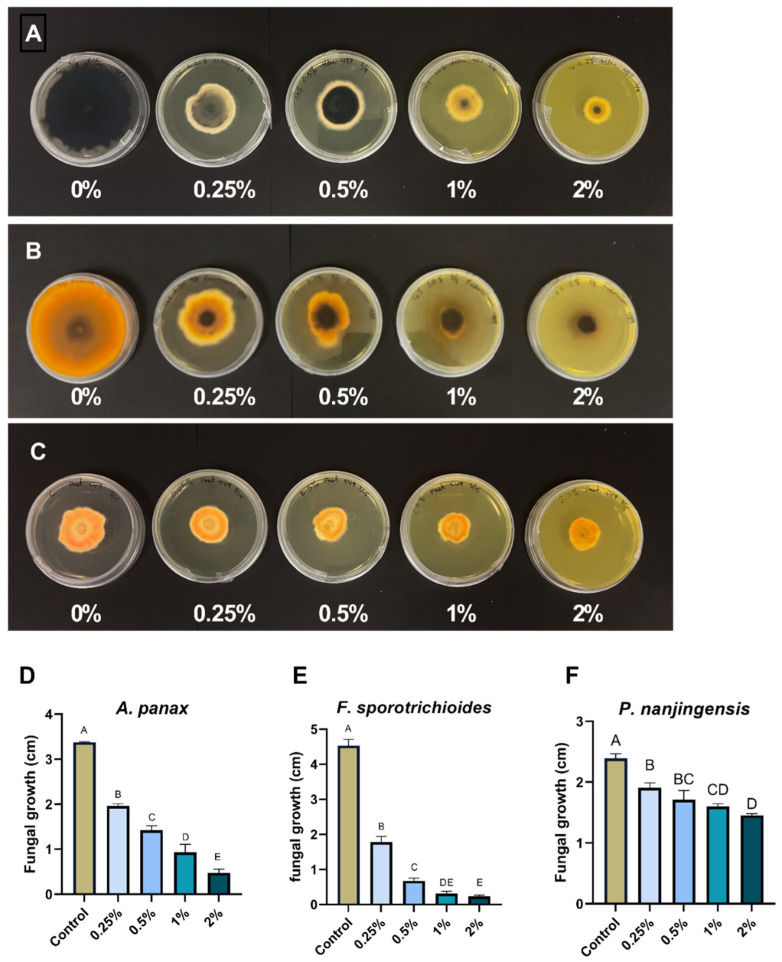
Anti-fungal effect of ethanolic extract of goldenseal root on the growth of (**A**,**D**) *A. panax*, (**B**,**E**) *F. sporotrichioides*, and (**C**,**F**) *P. nanjingensis*. Different letters on each bar represent values that are statistically different (*p* < 0.05).

The ethanolic extract of goldenseal root was assessed for its antifungal activity using an agar dilution assay. The ethanolic extract of goldenseal root significantly (*p* < 0.05) inhibited the growth of all three fungal isolates at all concentrations tested compared with the control (Figure 2). In general, the percentage of reduction in mycelial growth was dependent on the extract concentration in the medium. At the highest concentration tested (2%), the goldenseal root ethanolic extract showed an inhibition rate of 86.0%, 94.9%, and 39.1% against *A. panax*, *F. sporotrichioides*, and *P. nanjingensis*, respectively. At the lowest concentration tested (0.25%), goldenseal root ethanolic extract showed inhibition rates of 41.7%, 61.6%, and 20.2% against *A. panax*, *F. sporotrichioides*, and *P. nanjingensis*, respectively. In comparison, goldenseal root ethanolic extract exhibited higher potency in inhibiting *A. panax* and *F. sporotrichioides* than *P. nanjingensis*. At 0.5% concentration, the goldenseal root ethanolic extract inhibited the growth of both *A. panax* and *F. sporotrichioides* by >50%.

### 2.5. Effect of Aqueous Extracts of Goldenseal Root on the Growth of A. panax, F. sporotrichioides, and P. nanjingensis

**Figure 3:**
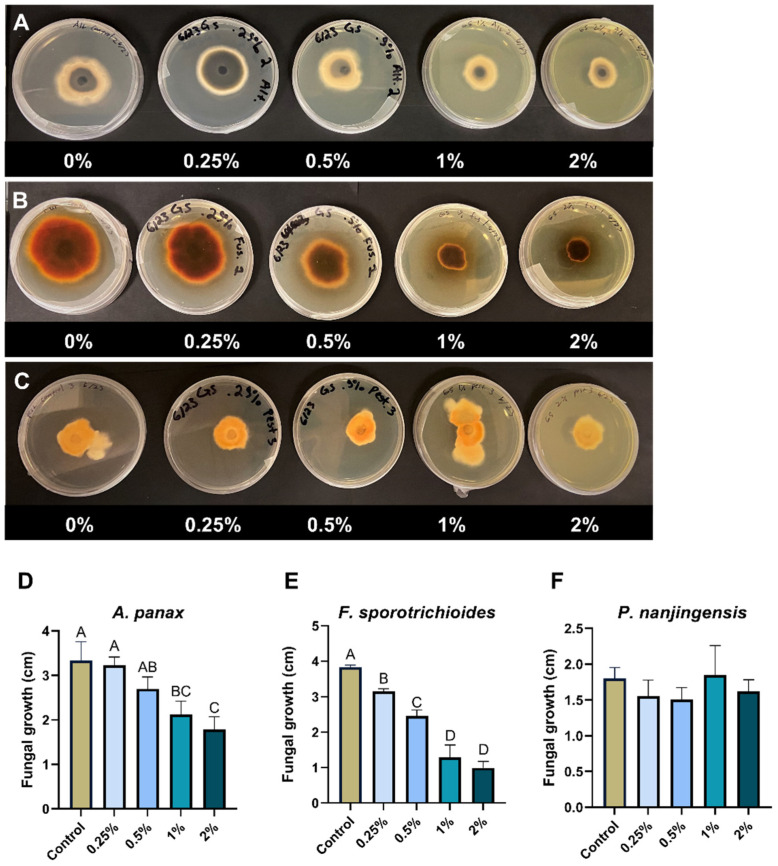
Anti-fungal effect of goldenseal root aqueous extract on the growth of (**A**,**D**) *A. panax*, (**B**,**E**) *F. sporotrichioides*, and (**C**,**F**) *P. nanjingensis*. Different letters on each bar represent values that are statistically different (*p* < 0.05).

In general, the percentage reduction in fungal growth showed dependency on the concentration of goldenseal root aqueous extracts. The goldenseal root aqueous extract significantly (*p* < 0.05) reduced the fungal growth of *F. sporotrichioides* at all concentrations tested (Figure 3B), with the highest inhibition rate of 76.1% at 2% concentration. At higher concentrations (1% to 2%), the goldenseal root aqueous extract also significantly reduced the growth of *A. panax* (*p* < 0.05) (Figure 3A). However, *P. nanjingensis* was not significantly reduced by the goldenseal root aqueous extract at any tested concentration (Figure 3C).

### 2.6. Effect of Goldenseal Leaf Ethanolic Extract on the Growth of A. panax, F. sporotrichioides, and P. nanjingensis

**Figure 4:**
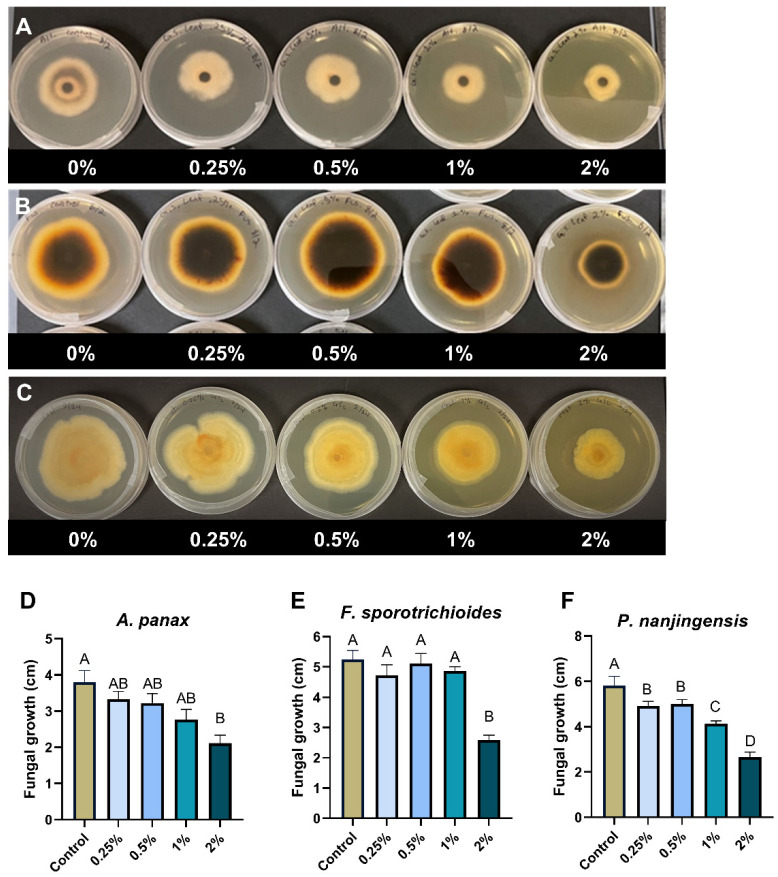
Anti-fungal effect of goldenseal leaf ethanolic extract on the growth of (**A**,**D**) *A. panax*, (**B**,**E**) *F. sporotrichioides*, and (**C**,**F**) *P. nanjingensis*. Different letters on each bar represent values that are statistically different (*p* < 0.05).

To explore the possible utilization of goldenseal leaves, we also tested the anti-fungal activity of the goldenseal leaf extract against *A. panax*, *F. sporotrichioides*, and *P. nanjingensis*. Our results showed that the goldenseal leaf extract was moderate in inhibiting the fungal isolates. Compared with the control, the goldenseal leaf extract showed significant inhibition against *A. panax*, *F. sporotrichioides*, and *P. nanjingensis* only at the highest concentration tested (2%), with inhibition rates of 44.4%, 50.8%, and 54.4%, respectively (Figure 4).

### 2.7. Effect of Goldenseal Root Extract on the Mycelial Morphology of Fungal Isolates

**Figure 5:**
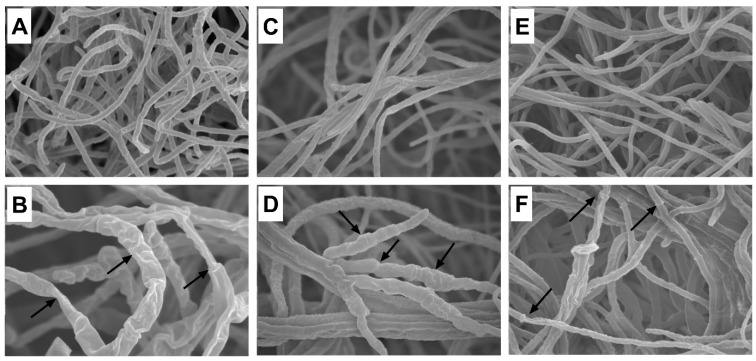
Scanning electron micrographs of *A. panax* grown on (**A**) PDA control plate and (**B**) in the presence of 1% goldenseal root ethanolic extract; *F. sporotrichioides* grown on (**C**) PDA control plate and (**D**) in the presence of 1% goldenseal root ethanolic extract; *P. nanjingensis* grown on (**E**) PDA control plate and (**F**) in the presence of 1% goldenseal root ethanolic extract. Arrows show abnormal mycelia.

The mycelial morphology of *A. panax*, *F. sporotrichioides*, and *P. nanjingensis* was observed under SEM. As shown in Figure 5, the mycelia in the untreated controls showed structural integrity and uniform distribution (Figure 5A, *A. panax*; Figure 5C, *F. sporotrichioides*; Figure 5E, *P. nanjingensis*). The morphology of the mycelium was round and plump, and it had a smooth surface. However, after treatment with 1% goldenseal root ethanolic extract, all three fungal species lost their normal morphological structure. The SEM micrograph of the mycelium of the fungi treated with goldenseal root ethanolic extracts displayed considerable morphological alterations. The mycelia were shrunken and wrinkled with warty surfaces. Some mycelia were empty and collapsed.

## 3. Discussion

In this study, three fungal isolates, *A. panax*, *F. sporotrichioides*, and *P. nanjingensis*, were successfully isolated and identified from the live tissues of diseased American ginseng collected from ginseng grower plots. While the isolates of *A. panax* and *F. sporotrichioides* in this study did not display symptoms of disease, potentially due to the inoculation method, it is noteworthy that these fungal species have been reported to be causative agents of leaf blight or root rot, which pose significant threats to the production of this crop [2,3,4,5,6,7]. To combat these pathogens, the use of goldenseal, a companion plant that grows alongside American ginseng in the same habitat, as a botanical pesticide was explored. The high alkaloid content of goldenseal makes it a potential option for preventing fungal pathogenic diseases, such as leaf blight and root rot, in American ginseng. The extraction methods of goldenseal were evaluated, and the anti-fungal activities of goldenseal extracts were investigated against the three fungal isolates that are associated with American ginseng.

In current cultivation practices, there are attempts to use goldenseal plants to make compost tea and apply it as a part of pest management. Compost tea is usually made with water as a solvent by macerating plant materials in water (Larry Harding and Ed Daniels, personal communication). However, most alkaloids are poorly soluble or insoluble in water but soluble in organic solvents such as ethanol [25]. Our study showed that both ethanol and deionized water yielded a high extraction rate (ranging from 22.6% to 34.6%) using sonication-assisted extraction. However, using ethanol as a solvent (ethanolic extract) yielded much higher alkaloid content than water (aqueous extract), and this is most likely due to the enhanced solubility of these alkaloids in organic solvents. This result suggested that ethanol is a better solvent for extracting alkaloids from goldenseal. Our study demonstrated that the common practice of making compost tea out of goldenseal using aqueous extraction methods is not as effective as ethanol extraction.

Although the antifungal activities of goldenseal extracts have been reported [20,21], this study provided evidence for the first time that goldenseal extracts have antifungal activity against plant pathogenic fungi. The antifungal effect of the goldenseal extract increased in direct relation to the alkaloid content level. The ethanolic extract of goldenseal roots showed notably higher inhibitory activity than that of the aqueous extract at all concentrations. At the lowest concentration tested (0.25%), the ethanolic extract of goldenseal roots showed significant inhibition against all three fungi, while the aqueous extract only showed significant inhibition against *F. sporotrichioides*. In addition, while the goldenseal extract did significantly (*p* < 0.05) reduce the mycelial growth of *P. nanjigensis*, the results were not as drastic as with *A. panax* and *F. sporotrichioides*. These data suggest that the efficacy of a goldenseal extract, when used as a fungicide, will likely be species-dependent.

When considering sustainability and the ease of harvest, utilizing the aerial (above-ground) part of a plant can be advantageous since harvesting the underground part often results in the destruction of the entire plant. In addition, the aerial part is usually more accessible than the underground part. With this in mind, we investigated the antifungal potential of the aerial parts of goldenseal, mainly the leaves. We found that the extraction rate of the goldenseal leaf extract was similar to that of the root extract. However, the alkaloid content, including berberine, canadine, and hydrastine, was reported to be higher in the underground part at various phenological stages and reproductive statuses compared to the aerial part [14]. According to our study, the goldenseal root extract showed greater potency compared to the leaf extract. The leaf extract only exhibited significant antifungal activity against three fungal isolates when used at a 2% concentration, which could be due to its lower alkaloid content compared to the root extract. However, our findings indicate that the aerial parts of goldenseal can still be effective if used at a higher concentration.

## 4. Materials and Methods

### 4.1. Plant Material

Dried goldenseal roots were purchased locally at Boring Herbs Inc., in Tennessee. Goldenseal leaves were picked at a private farm with the farm owner’s permission in Middle Tennessee in July 2022 and air-dried at ambient temperature after collection. Voucher specimens (GS202201R and GS202201L) were collected and deposited at the Middle Tennessee State University Herbarium. As noted in Zuiderveen et al. [14], the belowground structure of goldenseal consists of a rhizome (a modified subterranean stem) and multiple root fibers. We processed the roots and rhizomes together and referred to them collectively as “roots” in this manuscript.

### 4.2. Plant Extraction

Dried goldenseal roots and leaves were ground into a fine powder. Two grams of the fine powder was added to 15 mL of 70% ethanol. The solution was sonicated with a 6 mm probe for 30 min at 20.2 KH and then centrifuged for 5 min at 3500 rpm. The supernatant was transferred to a glass flask, and the solvent was evaporated at 50 °C for 30 min using a rotary evaporator. The residue was weighed and reconstituted with 70% ethanol to a stock concentration of 100 mg/mL. An aqueous extraction was performed following the same protocol as described above with the substitution of diH_2_O for 70% ethanol. The aqueous goldenseal extract was autoclaved to ensure all endophytic organisms were eliminated.

### 4.3. LC–MS Analysis

All solvents used in the LC–MS analysis were of spectroscopic grade (Avantor VWR, Radnor, PA, USA). Goldenseal extracts (both aqueous and 70% ethanolic) were filtered through a polyethersulfone (PES) membrane (0.45 µm, Avantor VWR) and diluted 1:100 in methanol, incorporating 1 µM chlorpropamide as an internal standard. Ultra-high-pressure (UHP) LC–MS data were collected using an Orbitrap Fusion Lumos Tribrid mass spectrometer (Thermo Scientific, Waltham, MA, USA) equipped with an electrospray ionization source coupled to a Vanquish UHPLC system (Thermo Scientific). Injections of 5 μL were subjected to reverse-phase UPLC on an Acquity BEH C18 column (150 × 2.1 mm, 1.7 μm particle size) (Waters Corp., Milford, MA, USA) that was maintained at 55 °C, with a flow rate of 100 μL/min. A 20 min binary solvent gradient was employed using solvent A (water with 0.1% formic acid) and solvent B (acetonitrile). The gradient started with an isocratic composition of 97:3 (A:B) for 1.0 min, transitioning linearly to 85:15 over 4 min and 5:95 over 11 min, followed by an isocratic hold at 5:95 for 2 min. The gradient then returned to starting conditions over 0.1 min and was held for 1.9 min. The positive ionization mode was employed, covering a full scan range of *m*/*z* 100–1000, with specific settings as follows: spray voltage, 3.5 kV; IT tube temperature, 275 °C; vaporizer temperature, 75 °C; sheath gas flow and auxiliary gas flow, 25 and 5 units, respectively.

### 4.4. Sampling and Fungal Isolates

The fungi used for the following bioassays, namely *A. panax*, *F. sporotrichioides*, and *P. nanjingensis*, were fungal isolates collected from infected American ginseng leaves or roots in Tennessee. Their initial symptoms were as follows: *A. panax* caused necrotic water-soaked symptoms on American ginseng leaves, with brown spots exhibiting yellow haloes observed on still green foliage. *F. sporotrichioides* led to darkly discolored ginseng roots, along with a wilted chlorotic to necrotic appearance in all above-ground parts. *P. nanjingensis* resulted in leaves exhibiting light brown spots confined within the leaf veins. 

All plant samples were surface-sterilized by submerging in 10% NaOCl_2_ for 60 s, followed by three washes with sterile water. Subsequently, the samples were plated on potato dextrose agar (PDA) in Petri dishes. Colonies were observed after incubation at 25 °C (light/dark: 12/12 h) for 10 days, and the colony morphologies were recorded. Fungal isolates were maintained on PDA plates at 25 °C. For long-term storage, the stock fungal cultures were transferred into 1.5 mL cryogenic vials with 30% glycerol solution and stored at −80 °C.

### 4.5. Fungal Isolate Identification

The fungal isolates were identified at Tennessee State University, Otis L. Floyd Nursery Research Center (NRC) plant pathology laboratory, McMinnville, Tennessee. The fungal isolates were identified via culturing, molecular, and pathogenicity tests. Detailed methods were described in Liyanapathiranage et al., 2023 [7]. Specifically, Total DNA was extracted from fungal colonies using the DNeasy PowerLyzer Microbial Kit (QIAGEN Sciences, Germantown, MD, USA). The ribosomal internal transcribed spacer (ITS) region, 18S small subunit (SSU), beta-tubulin (BT), translation elongation factors 1-α (EF1-α), and genetic markers were amplified using ITS1/ITS4, NS1/NS4 [26], T1/T2 [27], and EF1/EF2 [28], respectively. 

### 4.6. Fungal Pathogenicity Tests

For *A. panax*, the pathogenicity test involved inoculating six healthy 1-year-old American ginseng plants by placing a mycelial plug (5 mm in diameter) taken from a 7-day-old culture directly on the wounded leaf tissue. Six control plants were wounded but were not inoculated. For *F. sporotrichioides*, six healthy 1-year-old American ginseng plants were inoculated by placing mycelial plugs (5 mm in diameter) taken from a 7-day-old culture on 3 wounded roots per plant. Noncolonized PDA plugs were placed on the wounded roots of six plants and used as controls. For *P. nanjingensis*, six healthy 1-year-old American ginseng plants were spray-inoculated with a conidial suspension (1 × 106 conidia/mL) while six control plants were sprayed with sterile water.

All plants were covered with plastic bags and incubated in a greenhouse set at 21 °C to 23 °C, with 70% relative humidity and a 16 h photoperiod. After 48 h, bags were removed, and plants were maintained inside the greenhouse conditions mentioned above. Plants were observed for up to one month for symptom development. 

### 4.7. Agar Dilution Assay

The goldenseal extracts were assessed for antifungal activity using an agar dilution assay. Mycelial discs (8 mm plug) were sampled from the edges of actively growing fungal cultures (*A. panax*, *F. sporotrichioides*, and *P. nanjingensis*) and inoculated on PDA that was amended with 0%, 0.25%, 0.50%, 1.0%, and 2.0% goldenseal root or leaf extract. All three fungal isolates were tested against goldenseal extracts in triplicate and allowed to grow at 25 °C and 70% relative humidity. The antagonistic activity of goldenseal extract was determined after 7 days. The diameter of fungal growth was determined using ImageJ software (version 1.53p) [29] by averaging the vertical (V) and horizontal (H) diameters and subtracting the diameter of the initial mycelial plug (P) using the following formula:

$$\mathrm{Fungal}\;\mathrm{growth}\;(G)=\left(\frac{H+V}{2}\right)-P.$$

The inhibition percentage was calculated using the following formula:

Inhibition percentage (%) = (G1 − G2)/G1 × 100

where G1 is the colony area of the untreated fungus (control), and G2 is the colony area of fungus treated with various concentrations of goldenseal extracts.

### 4.8. Scanning Electron Microscopy (SEM) Imaging

Small sections (~3 mm) were excised from the edges of fungal growth and used for SEM. Samples were fixed in glutaraldehyde solution (2.5% in 0.1 M sodium cacodylate) for 1 h and then rinsed with 0.1 M sodium cacodylate twice and ultrapure water three times. Subsequently, samples were dehydrated in sequential steps using 30%, 50%, 70%, 95%, and 100% ethanol for 10 min each. This was followed by dehydration in ethanol:hexamethyldisilazane (HMDS) mixture (1:1) for 10 min and, finally, in 100% HMDS. Samples were air-dried overnight by allowing the 100% HMDS to evaporate completely in the hood. Samples were mounted for imaging, and images were taken with a Hitachi H3400 (Hitachi America, Santa Clara, CA, USA) at 15 KV.

### 4.9. Statistical Analysis

Normality was confirmed using the Shapiro–Wilk test. Analysis of variance (ANOVA) followed by Tukey’s multiple comparisons test was performed using GraphPad Prism version 9.4.0 (GraphPad software, San Diego, CA, USA).

## 5. Conclusions

The results of this study showed that ethanol is a suitable solvent for extracting the primary active ingredient, alkaloids, from goldenseal. The goldenseal root extract displayed strong antifungal activity against three fungal isolates that caused destructive diseases in American ginseng. The goldenseal root extract inhibited fungal growth by altering the mycelia of the fungal isolates. Collectively, these findings suggest that goldenseal extract has the potential to be used as a botanical pesticide to prevent leaf blight and root rot diseases in American ginseng caused by fungal pathogens in the genera *Alternaria*, *Fusarium*, and *Pestalotiopsis*.

## Figures and Tables

**Table 1:** Extraction rates of 70% ethanol and deionized water from dried goldenseal roots and leaves.

	Goldenseal Roots			Goldenseal Leaves		
	Raw Material	Extract	Yield	Raw Material	Extract	Yield
70% Ethanol	4000 mg	997 ± 144 mg	24.9 ± 3.6%	8000 mg	1810 mg	22.6%
H_2_O	4000 mg	1159 ± 129 mg	29.0 ± 3.2%	8000 mg	2770 mg	34.6%

## Data Availability

The data presented in this study are available in article and Supplementary Materials.

## Supplementary Materials

The following supporting information can be downloaded at: https://www.mdpi.com/article/10.3390/molecules29030556/s1. Table S1. Molecular identification of the fungal isolates.
