# Supplementary material for: Goldenseal (Hydrastis canadensis L.) Extracts Inhibit the Growth of Fungal Isolates Associated with American Ginseng (Panax quinquefolius L.)
Source: Molecules. 2024 Jan 23;29(3):556. doi: 10.3390/molecules29030556 (PMC10856682; doi:10.3390/molecules29030556)
Supplement: Supplementary file 1 [file molecules-29-00556-s001.zip › molecules-2748936-supplementary.pdf]

Supplementary Materials

Table S1. Molecular identification of the fungal isolates

Sample	Identification	ITS	EF -α	BT	SSU
Ginseng 550	*Pestalotiopsis* sp. *	100% to MK336517	100% to KU252416	100% to KU252503	-
Ginseng 549	*Pestalotiopsis* sp. *				
Ginseng 549	*Pestalotiopsis* sp. *				
Ginseng 550	*Pestalotiopsis* sp. *				
Ginseng 487	*Alternaria panax*	100% to MK534893	100% to LC480224	-	100% to KC584549
Ginseng 488	*Alternaria panax*				
Ginseng 489	*Fusarium sporotrichioides*	100% to MT635298	100% to MN553802	100% to AB587076	-

*Note: *Pestalotiopsis* sp. was further identified as *P. nanjingensis* based on morphology and molecular characteristics [6].
